# Dr. Daniel H. Brennan

**Published:** 1896-09

**Authors:** 


Dr. Daniel H. Brennan, of Albion, died at the Buffalo general hospital, August 11, 1896. It is stated that he had suffered ill-health for some time, and that about two weeks previous to his death he was brought to Buffalo for treatment. It was finally determined that an operation for appendicitis was necessary, which was made a few days before his death.

About two years ago he married Miss Barnett, of Albion, who survives him.

Dr. Brennan was a promising young physician, and his death occasioned great grief to his numerous and attached friends.

